# A CD209 ligand and a sialidase inhibitor differentially modulate adipose tissue and liver macrophage populations and steatosis in mice on the Methionine and Choline-Deficient (MCD) diet

**DOI:** 10.1371/journal.pone.0244762

**Published:** 2020-12-30

**Authors:** Darrell Pilling, Tejas R. Karhadkar, Richard H. Gomer

**Affiliations:** Department of Biology, Texas A&M University, College Station, TX, United States of America; The Ohio State University, UNITED STATES

## Abstract

Non-alcoholic fatty liver disease (NAFLD) is associated with obesity and type 2 diabetes and is characterized by the accumulation of fat in the liver (steatosis). NAFLD can transition into non-alcoholic steatohepatitis (NASH), with liver cell injury, inflammation, and an increased risk of fibrosis. We previously found that injections of either 1866, a synthetic ligand for the lectin receptor CD209, or DANA, a sialidase inhibitor, can inhibit inflammation and fibrosis in multiple animal models. The methionine and choline-deficient (MCD) diet is a model of NASH which results in the rapid induction of liver steatosis and inflammation. In this report, we show that for C57BL/6 mice on a MCD diet, injections of both 1866 and DANA reversed MCD diet-induced decreases in white fat, decreases in adipocyte size, and white fat inflammation. However, these effects were not observed in type 2 diabetic *db/db* mice on a MCD diet. In *db/db* mice on a MCD diet, 1866 decreased liver steatosis, but these effects were not observed in C57BL/6 mice. There was no correlation between the ability of 1866 or DANA to affect steatosis and the effects of these compounds on the density of liver macrophage cells expressing CLEC4F, CD64, F4/80, or Mac2. Together these results indicate that 1866 and DANA modulate adipocyte size and adipose tissue macrophage populations, that 1866 could be useful for modulating steatosis, and that changes in the local density of 4 different liver macrophages cell types do not correlate with effects on liver steatosis.

## Introduction

Non-alcoholic fatty liver disease (NAFLD) is part of a spectrum of chronic liver conditions that ranges from simple steatosis (abnormal accumulation of fat droplets within the hepatocytes) to hepatitis (accumulation of immune cells in the liver), that can result in non-alcoholic steatohepatitis (NASH), and ultimately fibrosis, cirrhosis, and liver failure [1–4]. Approximately one third of adults in industrialized nations have NAFLD, with 5–10% of these adults progressing to NASH, and a projected increase in NASH patients of 63% to 27 million cases by 2030 in the USA alone [4–6]. Up to 40% of individuals with NASH progress to advanced liver fibrosis and cirrhosis, and NASH patients are also at greater risk of developing hepatocellular carcinoma [7–9].

NAFLD and NASH are associated with type 2 diabetes, obesity, and metabolic syndrome (hyperglycemia, dyslipidemia, and systemic hypertension) [2, 9, 10]. These diseases lead to elevated circulating levels of lipids and carbohydrates which are converted to triglycerides and stored in hepatocytes. Excessive accumulation of lipids is cytotoxic, leading to hepatocyte cell stress including dysregulation of protein folding in the endoplasmic reticulum, inflammasome activation, production of reactive oxygen species (ROS), inflammatory cytokine production, and cell death [9, 11, 12]. These processes are inflammatory and lead to the activation of the tissue resident liver macrophages (Kupffer cells) as well as a recruitment of a variety of circulating immune cells (inflammation) [10, 13, 14]. Multiple insults appear to act to drive the progression from NAFLD to NASH and ultimately fibrosis [2, 15, 16].

NAFLD and NASH patients have elevated serum fatty acids which are released from adipose tissue and accumulate in the liver [9, 17–20]. This is due, in part, to a defective insulin response (insulin resistance) in adipocytes, with a reduced uptake of circulating lipids and increased release of stored triglycerides [15, 21]. In addition, increased numbers of adipose tissue and liver macrophages appears to correlate with progression from simple steatosis to NASH and fibrosis [22, 23]. This suggest that adipose tissue dysfunction is important in the generation of steatosis and liver inflammation in patients with NASH [21, 24]. Although many factors have been shown to be involved in NAFLD and NASH, how these molecules modulate inflammation and what is the role of the tissue resident liver macrophages (Kupffer cells) and recruited immune cells in driving or inhibiting NASH are yet to be clarified [25, 26]. In addition, it is still unclear if inflammation can be modulated to treat NASH, and if so which targets are likely to be the most effective [27, 28]. The methionine and choline-deficient (MCD) diet is a model that reflects the more serious complications of NASH including extensive liver steatosis, inflammation, and fibrosis [9, 29]. Methionine is an essential amino acid and is an intermediate in the synthesis of S-adenosylmethionine (SAM) and glutathione (GSH), two important antioxidants [20]. Choline is the precursor for phosphatidylcholine, the main component of cell membranes, and also necessary for very low-density lipoprotein (VLDL) synthesis and the export of triglycerides by hepatocytes [30]. Methionine deficiency thus predisposes to mitochondrial oxidative stress, resulting in cellular dysfunction and cell death, and choline deficiency leads to accumulation of triglycerides within cells, resulting in steatosis [20, 31]. The MCD diet mimics the steatosis and liver inflammation associated with NASH, without the systemic changes and weight gain associated with obesity [18, 20, 32]. To overcome some of these issues, *db/db* mice, which carry a point mutation in the leptin receptor and are obese, insulin resistant, and develop type 2 diabetes, when fed the MCD diet do progress from simple steatosis to steatohepatitis and fibrosis, in the context of metabolic syndrome [33, 34].

Pentraxins are a family of highly conserved secreted proteins that regulate the innate immune system and have a profound effect on the development of inflammation [35–37]. The pentraxin serum amyloid P (SAP; also called PTX2) reduces neutrophil activation and recruitment [38–40], regulates the differentiation of monocytes into macrophages [37, 41], and induces macrophages to secrete the anti-inflammatory cytokine IL-10 [42–44]. Plasma SAP levels are significantly lower in patients with NAFLD compared to non-NAFLD controls, and decline further in patients with advanced disease [45]. SAP inhibits inflammation and fibrosis and promotes disease resolution by activating the high affinity IgG receptor Fcγ receptor I (FcγRI; CD64) [42, 46–49] and the dendritic cell-specific intercellular adhesion molecule-3-grabbing non-integrin (DC-SIGN; CD209) [47, 50–52]. CD209 activation by a synthetic ligand [53] can mimic SAP effects on neutrophils, monocytes, and macrophages [47]. SAP and a CD209 ligand can inhibit high fat diet-induced adipose tissue and liver inflammation and steatosis in mice, and SAP can inhibit high fat diet-induced atherosclerosis [54, 55].

Sialic acids are often found as the distal terminal sugar on the oligosaccharide chains of glycoconjugates such as glycoproteins. Sialidases (also called neuraminidases) are enzymes which remove this sialic acid from glycoconjugates [56]. In mammals, there are 4 sialidases, NEU 1–4 [57]. N-Acetyl-2,3-dehydro-2-deoxyneuraminic acid (DANA) inhibits mammalian sialidases [58]. We previously found that injections of DANA or the lack of NEU3 both attenuate bleomycin-induced lung fibrosis in mice [59, 60].

As liver steatosis, inflammation, and fibrosis are hallmarks of NAFLD/NASH, and since injections of a CD209 ligand and DANA both inhibit inflammation and fibrosis, we examined whether injections of DANA or a CD209 ligand could inhibit MCD diet-induced liver inflammation and steatosis and adipose tissue loss in mice.

## Materials and methods

### Mouse model of obesity

This study was carried out in strict accordance with the recommendations in the Guide for the Care and Use of Laboratory Animals of the National Institutes of Health. All procedures were done with specific approval of the Texas A&M University institutional animal care and use committee (TAMU IACUC# 2018–0178). All procedures were performed under anesthesia (4% isoflurane in oxygen) and all efforts were made to minimize suffering. 8 week old male C57BL/6 mice (#000664; Jackson Laboratory, Farmington, CT) and spontaneous diabetic *db/db* mice (#000697; *Lepr*^*db*^, Jackson Labs) were maintained on standard rodent chow (15% kcal fat, Teklad 8604, Envigo, Madison WI). Mice were transferred to methionine and choline deficient (MCD) diet (A02082002BR; Research Diets, New Brunswick, NJ) or control methionine and choline sufficient diet (A02082003BY; Research Diets) at day 0. C57BL/6 mice were maintained on MCD and control diets for 21 days and *db/db* mice were maintained on MCD and control diets for 28 days. Mice were randomly assigned to dietary and treatment groups. Mice were housed with a 12-hr/12-hr light-dark cycle with free access to food and water. All procedures were performed between 09:00 and noon. A total of 16 C57BL/6 and 17 *db/db* mice were used in this study.

Intraperitoneal (i.p.) injections of 20 mM sodium phosphate pH 7.2 buffer only, DANA (N-Acetyl-2,3-dehydro-2-deoxyneuraminic acid; EMD-Millipore, Burlington, MA) at 10 mg/kg in sodium phosphate buffer, or CD209 ligand “1866” (#5931866, ChemBridge Corporation, San Diego, CA) at 0.1 mg/kg in sodium phosphate buffer, were given every 48 hours, as described previously [47, 54, 59, 60]. For glucose tolerance tests (GTT), mice were fasted for 16 hours before i.p. injections of 1.5 g/kg glucose (Amresco, Solon, OH) in PBS, as described previously [54]. Blood glucose levels were measured before glucose administration (0 minutes), and at 20, 40, 60, 90, and 120 minutes after the injection using commercial blood glucose test strips (CVS Pharmacy, Woonsocket, RI). Mice were euthanized by asphyxiation with CO_2_ [61], and no anesthesia was used during euthanasia.

### Histology and antibody staining

After mice were euthanized, blood was collected from the abdominal aorta and chilled on ice. After 30 minutes, the blood was clarified by centrifugation at 10,000 × g for 5 minutes at 4°C to isolate serum, which was then stored at -80°C. Organs including epididymal white adipose and inter-scapular brown adipose tissue, liver, spleen, lungs, and kidneys were weighed before processing. Pieces of adipose tissue and liver were snap frozen in liquid nitrogen and stored at -80˚C; embedded in OCT compound (VWR, Radnor, PA), frozen, and stored at -80˚C; or were fixed in Zn-buffered formalin solution (0.1% ZnSO_4_; 3.8% formaldehyde; VWR, Radnor, PA) for 2 days on ice, and then placed in 10% and then 30% sucrose solution in PBS for 2 days each on ice. Fixed tissues were then kept in 70% ethanol at room temperature until paraffin processing and sectioning at 5 μm.

Before antibody staining, fixed tissue sections were de-paraffinized, and antigens were retrieved using 10 mM sodium citrate, pH 6.0 at 98°C for 20 minutes, as described previously [62]. As described previously [40, 54, 59], sections of adipose and liver tissue were stained with 5 μg/ml antibodies against Mac2 (rat mAb, clone M3/38, BioLegend, San Diego, CA) to detect recruited and tissue macrophages, F4/80 (rabbit mAb, D2S9R, Cell Signaling Technology, Danvers, MA) to detect tissue resident macrophages, CLEC4F (goat Ab, AF2784, Novus Biologicals, Littleton, CO) to specifically detect Kupffer cells, CD64 (rabbit mAb 50086-R001, SinoBiological, Wayne, PA) to detect FcγRI expression, and MRP8 (goat Ab, AF3059, Novus Biologicals) to detect neutrophils. Secondary F(ab’)_2_ biotin-conjugated donkey anti-rat, anti-goat, or anti-rabbit antibodies were from Jackson ImmunoResearch (West Grove, PA) or Novus Biologicals, and biotinylated antibodies were revealed with streptavidin-conjugated-alkaline phosphatase staining (Vector Laboratories, Burlingame, CA). Sections were counter stained with hematoxylin. To determine the amount of steatosis (accumulation of fat in the cells of the liver) and adipocyte size, paraffin embedded tissue sections were stained with hematoxylin and eosin, as described previously [40, 54]. To determine liver fibrosis, sections were stained with sirius red, which detects collagen [63]; as described previously [54, 64].

### Cytokine and serum protein quantification

Serum cytokines were measured with a 13-plex LEGENDplex Mouse Inflammation Panel kit (BioLegend) following the manufacturer’s instructions using an Accuri C6 flow cytometer (Accuri C6; BD Biosciences, San Jose, CA). Data were analyzed using LEGENDplex data analysis software version 8.0 (BioLegend), and the concentration of proteins was calculated from standard curves. Serum alanine transaminase (ALT), aspartate aminotransferase (AST), triglyceride, and cholesterol levels were measured with kits following the manufacturer’s instructions (Cayman Chemical, Ann Arbor, MI).

### Image quantification

Tissue sections stained with antibodies, sirius red, or hematoxylin and eosin were imaged with a Nikon Eclipse Ti2 microscope (Nikon Instruments, Melville, NY) and analyzed with ImageJ2 software version 1.53e [65]. The percentage area of tissue stained with was quantified as a percentage of the total area of the tissue, as described previously [40, 54, 59, 64]. Adipocyte size was calculated using the ImageJ plug-in Adiposoft version 1.16 (Imaging Unit of the Center for Applied Medical Research (CIMA), University of Navarra, Pamplona, Spain) [66].

### Statistical analysis

Statistical analysis was performed using Prism v7 (GraphPad Software, La Jolla, CA). Statistical significance between two groups was determined by t test, or between multiple groups using analysis of variance (ANOVA) with Dunnett’s or Sidak’s post-test, and significance was defined as p<0.05.

## Results

### 1866 and DANA reverse MCD diet-induced reduction in white fat in C57BL/6 mice tissue and reduce liver weight in *db/db* mice

**Fig 1:**
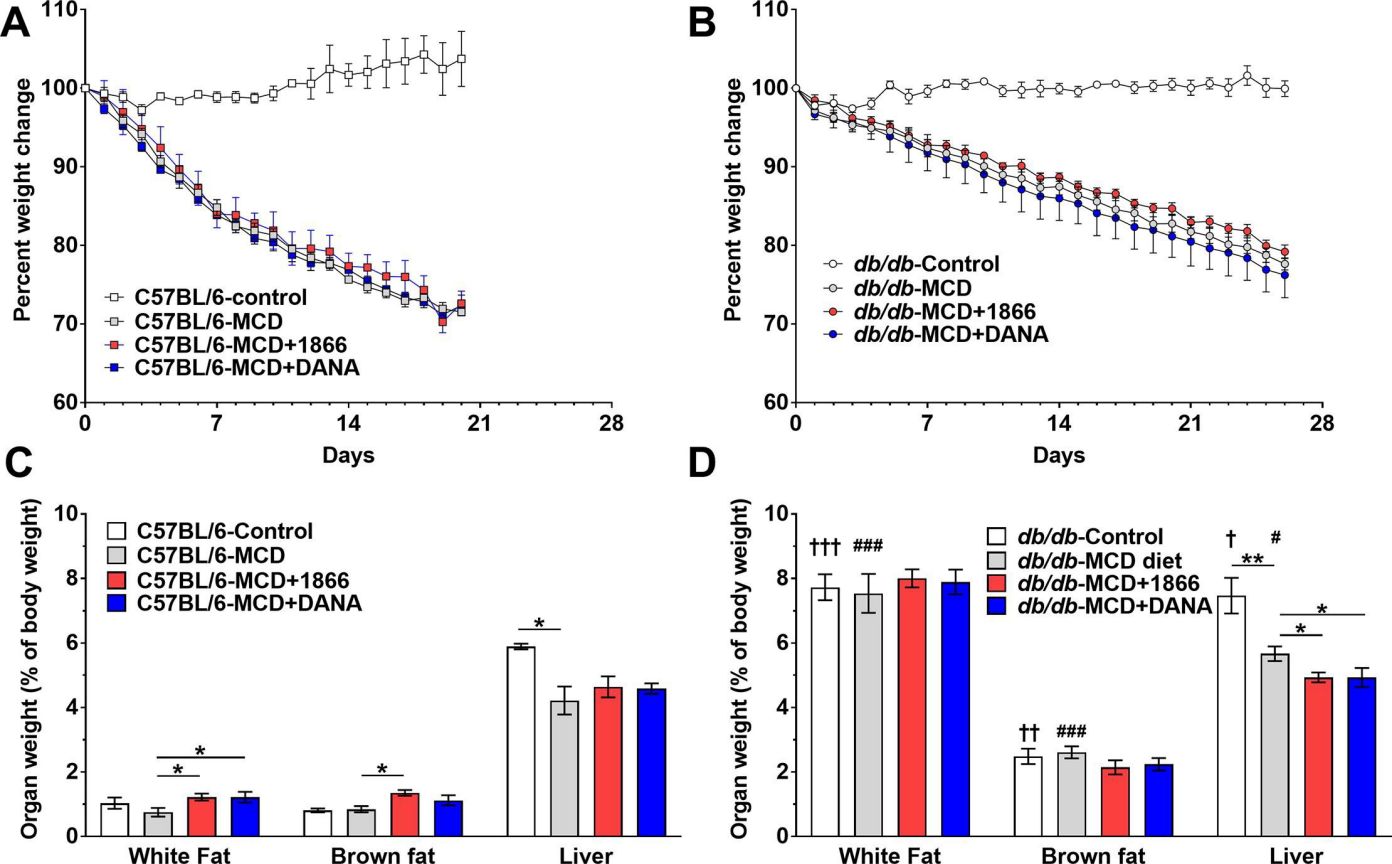
1866 and DANA effects on MCD diet-induced changes in adipose tissue and liver weights. C57BL/6 and *db/db* mice were transferred to methionine and choline sufficient (Control) or methionine and choline deficient (MCD) diet at day 0 and were injected every 48 hours with buffer, 1866, or DANA. Graphs show body weights of **A)** C57BL/6 and **B)** *db/db* mice weighed up until the day mice were fasted for a glucose tolerance test and then euthanized. At day 21 for **C)** C57BL/6 or **D)** 28 days for *db/db* mice, post-euthanasia epididymal white fat, brown fat, and livers were weighted. Values are mean ± SEM, n = 3 for C57BL/6 on regular diet, n = 4 for *db/db* on regular diet, n = 5 for C57BL/6 and *db/db* mice on the MCD diet, and n = 4 for mice on the MCD diet and then treated with 1866 or DANA. * indicates p < 0.05 and **p < 0.01 (one-way ANOVA, Sidak’s test). † indicates p < 0.05, †† p< 0.01, and ††† p<0.001 comparing C57BL/6 and *db/db* mice on control diet (t-test). # indicates p < 0.05, ### p<0.001 comparing C57BL/6 and *db/db* mice on MCD diet (t-test).

DANA and the DC-SIGN ligand 1866 both inhibit inflammation and fibrosis in multiple animal models [47, 54, 59, 60]. Although NASH is thought of as an inflammatory process of the liver, NASH and the MCD diet model also induce changes in adipose tissue [8, 9, 18, 19]. Therefore, we assessed whether DANA or 1866 could attenuate MCD diet-induced weight loss [20] and changes in adipose tissue. DANA and 1866 did not significantly affect weight loss of either C57BL/6 or *db/db* mice on the MCD diet (Fig 1A and 1B). At day 21 on the MCD diet, C57BL/6 mice had reduced liver weight (Fig 1C). On the MCD diet, both DANA and 1866 increased white fat weight, 1866 increased brown fat weight, but neither treatment significantly affected liver, spleen, heart, kidney, or lung weights (Fig 1C and S1A Fig).

On the MCD diet, C57BL/6 mice lost more weight (1.43 ± 0.07%/day) than *db/db* mice (0.78 ± 0.01%/day) (linear regression; p < 0.001, F test) (Fig 1A and 1B). As expected [20, 67], compared to C57BL/6 mice, *db/db* mice fed either control or MCD-diet had higher adipose tissue and liver weights (Fig 1C and 1D). The MCD-diet caused *db/db* mice to have lower liver weights (Fig 1D), but did not significantly affect adipose tissue, spleen, heart, kidney, or lung weights (Fig 1D and S1B Fig). For *db/db* mice on the MCD diet, both 1866 and DANA reduced liver weight, but neither 1866 or DANA significantly affected adipose tissue, spleen, heart, kidney, or lung weights (Fig 1C and S1B Fig). Similar statistical significance was determined when the absolute body and organ weights were assessed (S1C and S1D Fig). There were no significant differences between the weights of other organs. These data indicate that injections of DANA and 1866 can modulate adipose tissue weights in C57BL/6 MCD diet mice and liver weights in *db/db* MCD diet mice.

### DANA and 1866 have little effect on MCD diet-induced changes in systemic glucose levels

**Fig 2:**
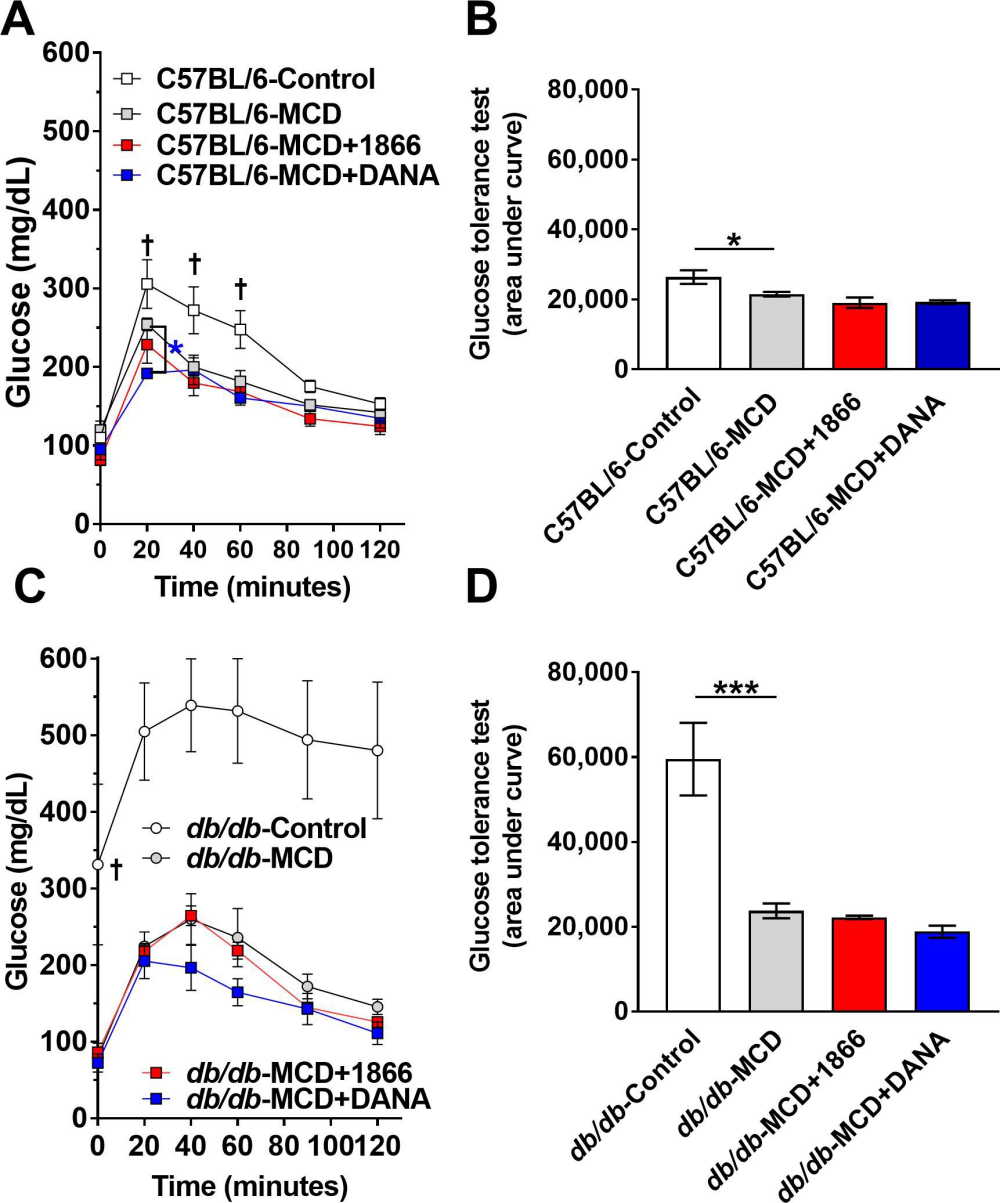
1866 and DANA have little effect on MCD diet-induced changes in systemic glucose levels. C57BL/6 and *db/db* mice were transferred to methionine and choline sufficient (Control) or methionine and choline deficient (MCD) diet at day 0 and they were injected every 48 hours with PBS buffer, 1866, or DANA. At **A)** 20 days C57BL/6 and **C)** 27 days *db/db* mice were fasted overnight and then received an i.p. injection of glucose (1.5 g/kg body weight), and tail vein blood samples were assessed for glucose at the indicated times. **B and D)** Glucose tolerance over 120 minutes was assessed by area under the curve analysis. In **A**, † indicates p < 0.05 comparing C57BL/6 mice on control and MCD diet (t-test), * indicates p < 0.05 comparing C57BL/6 mice on MCD and MCD+DANA (t-test). In **C**, † indicates p < 0.05 comparing *db/db* mice on control and MCD diet (t-test). In **B** and **D**, * indicates p < 0.05 and ***p < 0.001 (one-way ANOVA, Sidak’s test).

The MCD diet or the *db/db* mutation lead to systemic metabolic dysregulation, and the *db/db* mutation leads to type 2 diabetes [33, 67, 68]. Compared to C57BL/6 mice on the control diet, mice on the MCD diet treated with either 1866, DANA, or buffer injections did not have a significant difference in fasting glucose levels (t = 0 minutes), (Fig 2A). Compared to C57BL/6 mice on a control diet, MCD diet-fed mice had decreased glucose levels at 20, 40, and 60 minutes after a glucose injection (Fig 2A and 2B). MCD diet fed C57BL/6 mice injected with DANA had lower glucose levels at 20 minutes compared to control MCD mice (Fig 2A). Using area under the curve (AUC) analysis, compared to C57BL/6 mice on control diet, MCD diet fed C57BL/6 mice had lower total glucose levels (Fig 2B). Injections of 1866 or DANA had no significant effect on AUC glucose levels (Fig 2B).

As expected [68], compared to C57BL/6 mice, *db/db* mice on the control diet had higher fasting glucose levels (Fig 2A and 2C). Compared to *db/db* mice on a control diet, MCD diet-fed *db/db* mice had lower glucose levels, both at t = 0 and after the glucose injection (Fig 2C and 2D). There was no significant effect of 1866 or DANA on blood glucose levels after glucose injection on MCD diet *db/db* mice (Fig 2C and 2D). These results suggest that 1866 has no significant, and DANA has a very slight, effect on glucose levels in the C57BL/6 or *db/db* MCD diet models.

### 1866 and DANA decrease crown-like structures in *db/db* white adipose tissue

**Fig 3:**
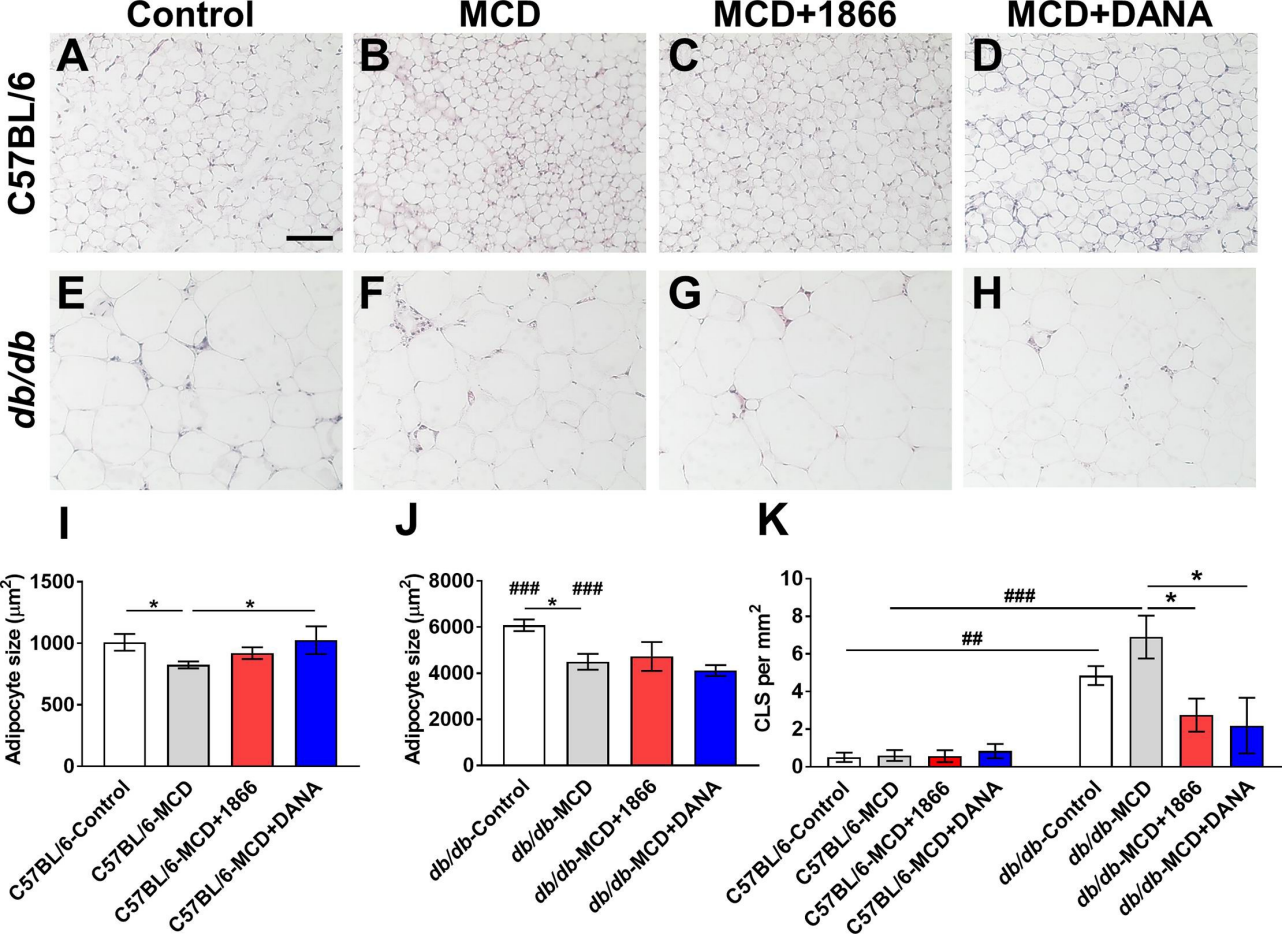
1866 and DANA effects on MCD-induced changes in white adipose tissue. **A-H)** Representative images of epididymal white fat sections of **A-D)** C57BL/6 mice or **E-H)** or *db/db* mice, on **A and E)** control diet, **B and F)** MCD diet, **C and G)** MCD+1866, or **D and H)** MCD + DANA were stained with hematoxylin and eosin. Images are representative of three to five mice per condition. Bar is 0.1 mm. Average adipocyte area was calculated for **I)** C57BL/6 and **J)** *db/db* mice. **K)** Crown like structures (CLS) in white fat were counted for C57BL/6 and *db/db* mice. Values are mean ± SEM, n = 3–5 mice per group. * indicates p < 0.05 (one-way ANOVA, Sidak’s test) or ## p < 0.01, ### p< 0.001 indicates comparing C57BL/6 and *db/db* mice on control or MCD diet (t test).

The MCD diet leads to increased lipase activity in adipose tissue and a reduction in adipocyte size, whereas loss of leptin receptor signaling in the *db/db* mouse leads to an increase in adipocyte size, leading to mechanical and hypoxic stress, elevated lipolysis, altered cytokine production, and reduced production of leptin and adiponectin [33, 67, 69]. Compared to white adipose tissue from C57BL/6 mice on the control diet (Fig 3A and 3I), C57BL/6 mice on the MCD diet had smaller adipocytes, and DANA but not 1866 reversed this (Fig 3B–3D and 3I). Compared to control diet C57BL/6 mice, control diet *db/db* mice had larger adipocytes (Fig 3A, 3E and 3J). The MCD diet decreased *db/db* adipocyte size (Fig 3E, 3F and 3J), and 1866 or DANA did not affect this (Fig 3G, 3H and 3J).

Increased numbers of inflammatory cells such as macrophages in adipose tissue in obesity and the MCD diet model may contribute to both local adipocyte tissue dysfunction and drive systemic inflammation [70, 71]. Crown-like structures (CLS) are aggregates of macrophages that are thought to scavenge lipid droplets and dead adipocytes, and the number of CLS correlates with systemic insulin resistance in obese patients [72–74]. For C57BL/6 mice, the MCD diet with or without 1866 or DANA injections did not significantly affect numbers of CLS (Fig 3K). Compared to C57BL/6 mice, *db/db* mice on both control and MCD diet had increased numbers of CLS (Fig 3K). For *db/db* mice on the MCD diet, 1866 or DANA decreased the number of CLS (Fig 3F–3H and 3K). These data suggest that injections of 1866 or DANA decrease macrophage accumulation in white adipose tissue in *db/db* mice on the MCD diet.

### 1866 and DANA decrease macrophages in adipose tissue of C57BL/6 mice on the MCD diet

**Fig 4:**
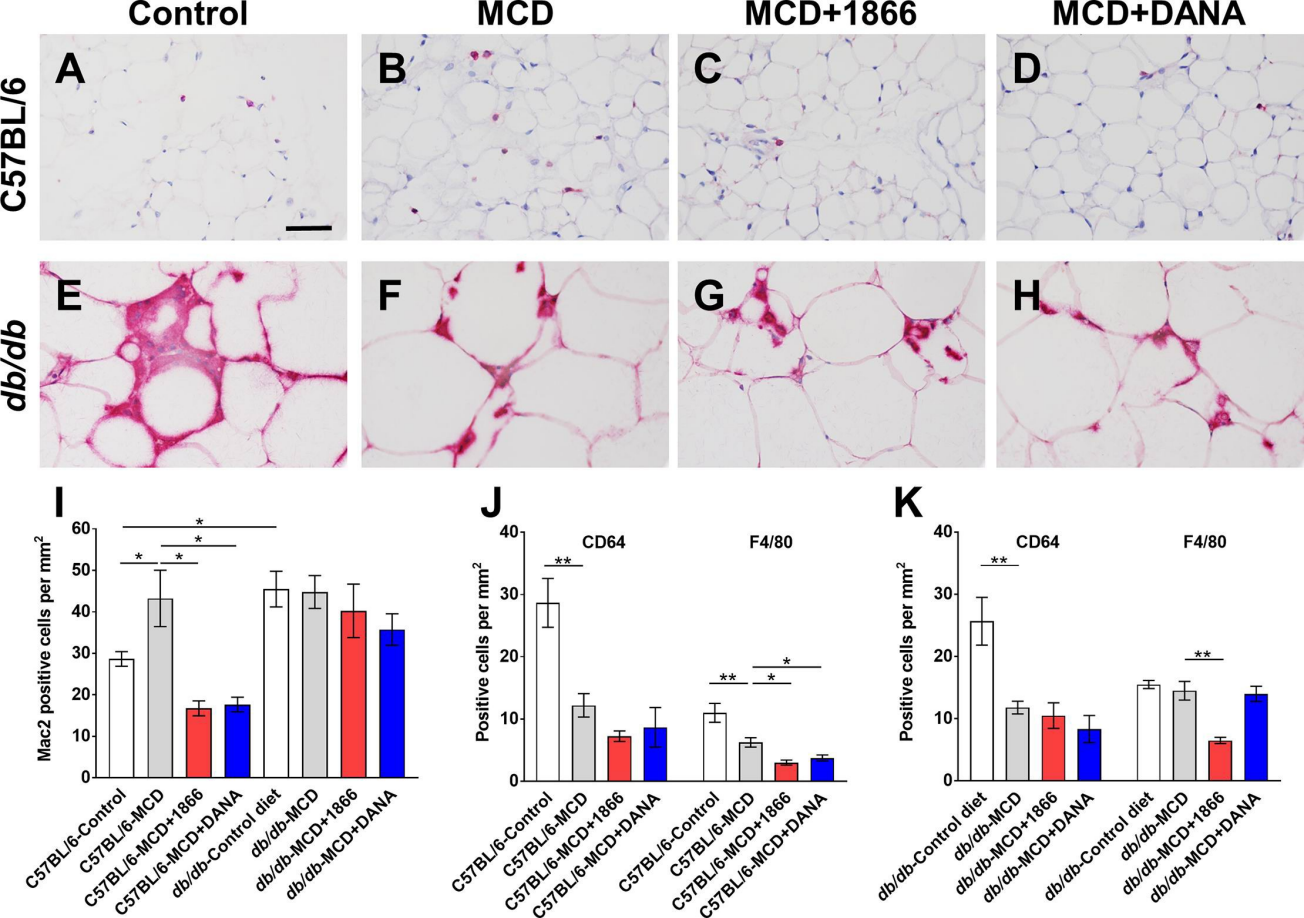
1866 and DANA effects on macrophage populations in adipose tissue. **A-H)** Representative images of epididymal white fat sections of **A-D)** C57BL/6 mice or **E-H)** or *db/db* mice, on **A and E)** control diet, **B and F)** MCD diet, **C and G)** MCD+1866, or **D and H)** MCD + DANA were stained with anti-Mac2 antibodies. Bar is 0.05 mm. **I)** Quantification of Mac2 positive cells, **J)** Quantification of CD64 positive cells and **K)** F4/80 positive cells. Values are mean ± SEM, n = 3–5 mice per group. * indicates p < 0.05, ** p < 0.01 (one-way ANOVA, Sidak’s test).

Mac2 (also known as Galectin-3) is a cell surface lectin receptor present on monocytes and macrophages and is released following cellular activation, and elevated levels of adipose tissue and serum galectin-3 are associated with obesity and NASH [75–82]. For C57BL/6 mice, the MCD diet increased numbers of Mac2 positive cells in the adipose tissue, and 1866 or DANA reversed this (Fig 4A–4D and 4I). Compared to C57BL/6 mice, *db/db* mice on the control diet had increased numbers of Mac2 positive cells in the adipose tissue (Fig 4A, 4E and 4I). For *db/db* mice, the MCD diet with or without 1866 or DANA did not significantly affect Mac2 positive cells numbers (Fig 4F–4H and 4I).

For C57BL/6 mice, the MCD diet decreased numbers of CD64 and F4/80 positive tissue resident adipose macrophages [79–82] (Fig 4J). 1866 or DANA had no significant effect on CD64 positive cells, but 1866 and DANA further reduced F4/80 positive cells in adipose tissue of MCD diet C57BL/6 mice (Fig 4J). For *db/db* mice, as with C57BL/6 mice, the MCD diet decreased numbers of CD64 positive cells, and 1866 or DANA had no significant effect on this (Fig 4K). The MCD diet did not significantly affect *db/db* F4/80 positive cell numbers, and 1866 reduced these numbers (Fig 4K). These data suggest that for C57BL/6 mice, MCD diet-induced increases in white adipose tissue recruited macrophage numbers can be attenuated by injections of 1866 or DANA.

### 1866 reduces MCD diet-induced liver steatosis in *db/db* mice

**Fig 5:**
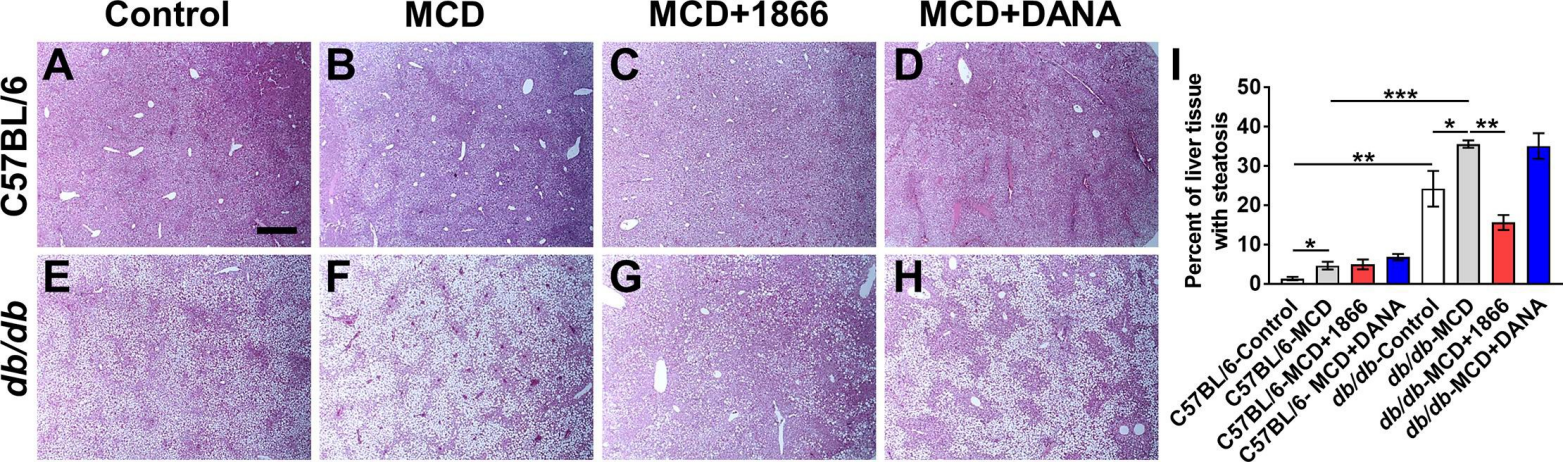
1866 reduces MCD diet-induced liver steatosis in *db/db* mice. **A-D)** C57BL/6 and **E-H)** *db/db* mice on **A and E)** control diet, **B and F)** MCD diet, **C and G)** MCD+1866, or **D and H)** MCD + DANA. Images show representative liver sections stained with hematoxylin and eosin. Bar is 0.5 mm. **I)** Quantification of steatosis in liver sections. Values are mean ± SEM, n = 3–5 mice per group. * indicates p<0.05, ** p < 0.01, *** p < 0.001 (one-way ANOVA, Sidak’s test).

**Fig 6:**
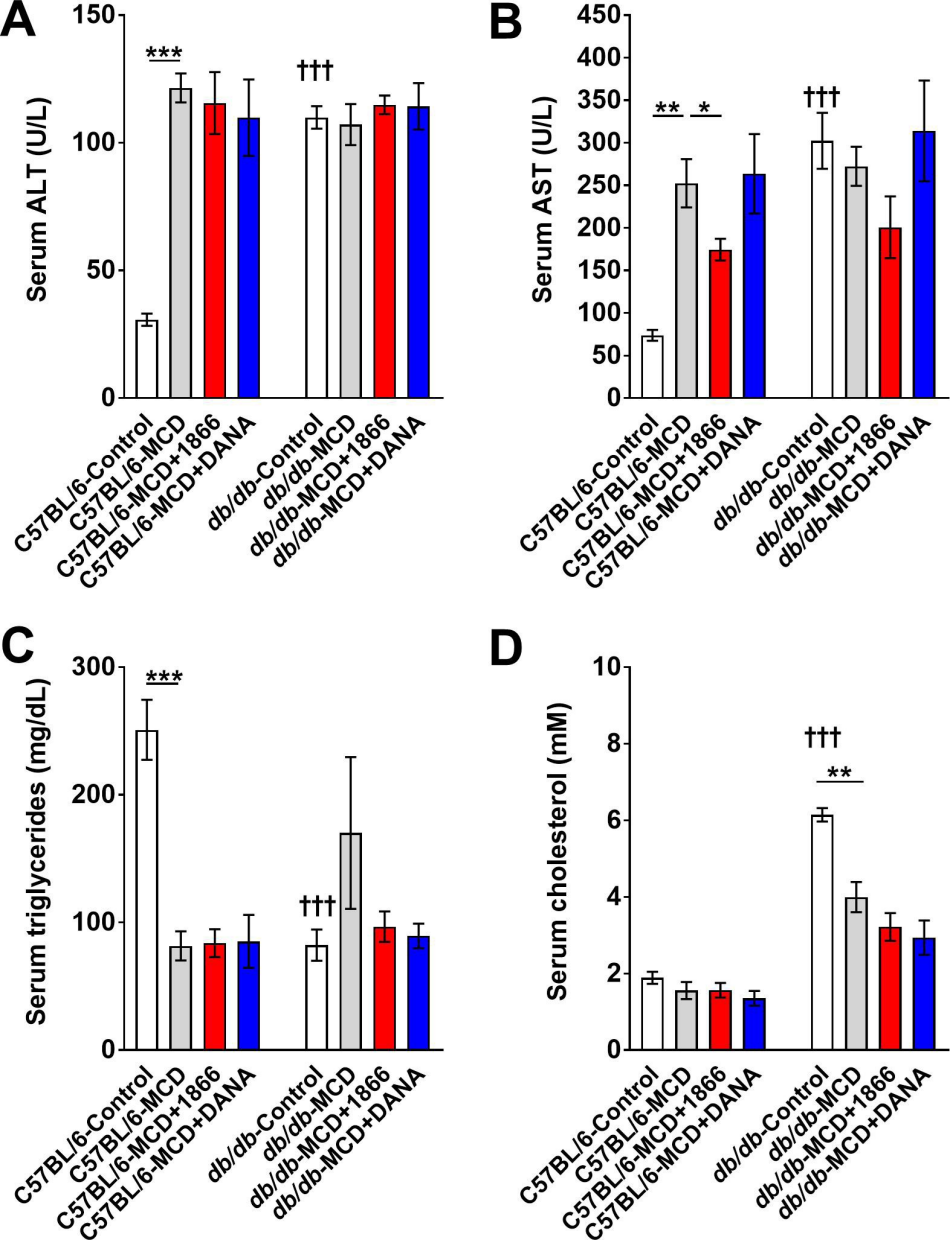
Quantification of serum ALT, AST, and lipids. Serum from C57BL/6 and *db/db* mice was assessed for **A)** ALT, **B)** AST, **C)** triglycerides or **D)** cholesterol. * indicates p<0.05, ** indicates p < 0.01, *** p < 0.001 (one-way ANOVA, Sidak’s test). ††† indicates p<0.001 comparing C57BL/6 and *db/db* mice on control diet (t-test).

Humans with obesity, NAFLD, or NASH, *db/db* mice, and mice on the MCD diet tend to show increased liver steatosis (accumulation of fat droplets within the hepatocytes) [1, 20, 26]. As expected, we found that compared to C57BL/6 mice, *db/db* mice on control diet had increased liver steatosis (Fig 5A, 5E and 5I), increased serum ALT, AST, and cholesterol levels, and reduced serum triglycerides (Fig 6A–6D). The MCD diet increased steatosis for both strains (Fig 5A, 5B, 5E, 5F and 5I). 1866 and DANA injections had no significant effect on steatosis in MCD diet C57BL/6 mice (Fig 5I), but 1866 reduced serum AST levels (Fig 6B). 1866 reduced steatosis in MCD diet *db/db* mice (Fig 5F, 5G and 5I) without affecting serum ALT, triglycerides, or cholesterol, although there was a trend at reduction in AST (p = 0.057, t test) (Fig 6A, 6C and 6D). These data suggest that 1866 can reverse some of the effects of MCD diet on liver steatosis in *db/db* mice.

MCD diets can lead to elevated inflammatory cytokines in adipose tissue, liver, and serum [20, 83–85]. For a variety of serum cytokines, there was no significant differences between C57BL/6 and *db/db* mice, or between control and MCD diet, or treatment with 1866 or DANA (S2 Fig).

### 1866 and DANA reduce MCD diet-induced changes in liver inflammation and fibrosis

**Fig 7:**
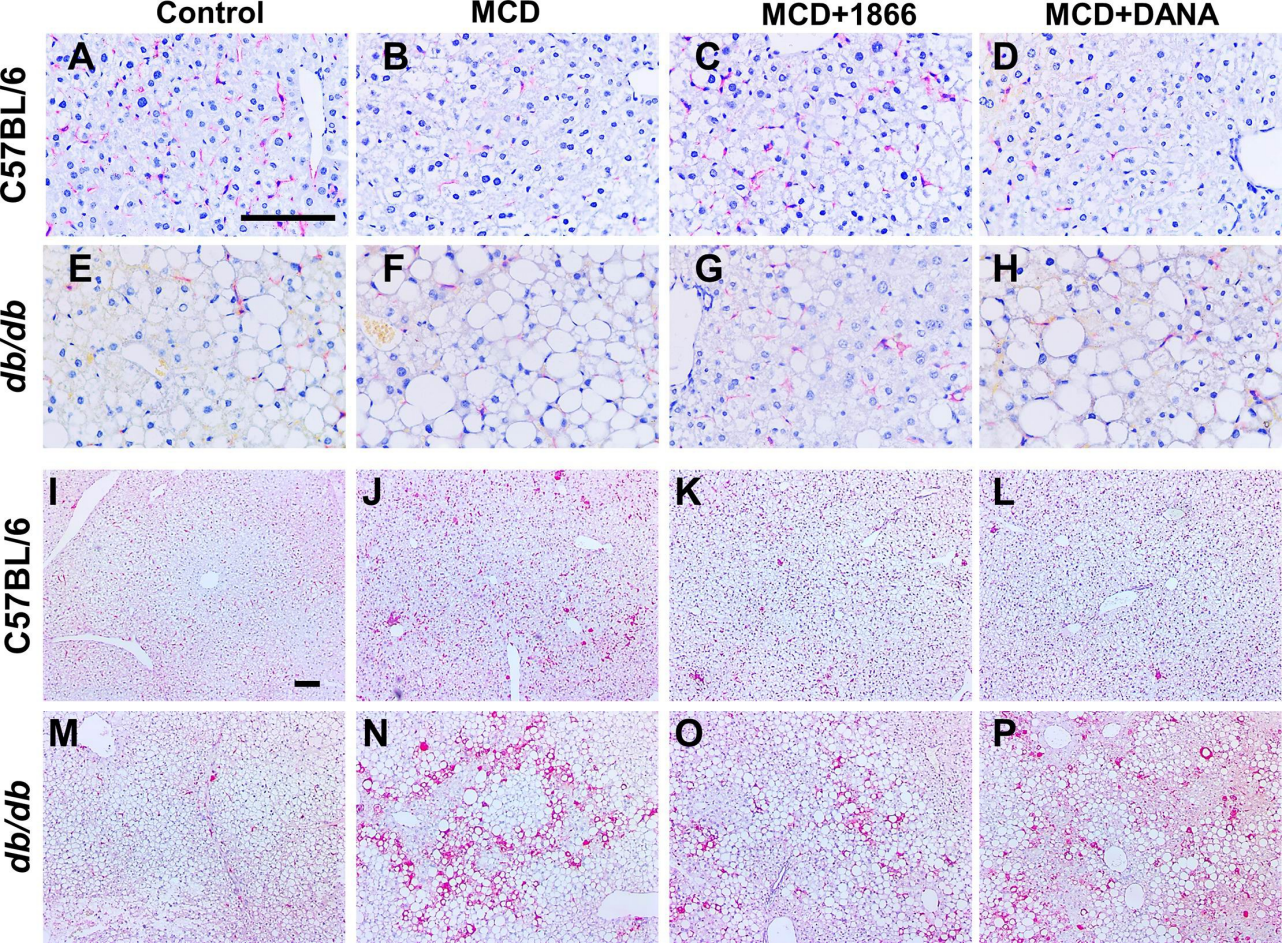
MCD diet-induced changes in liver macrophages. Representative images of liver sections stained with anti-CLEC4F antibodies from **A-D)** C57BL/6 mice or **E-H)** or *db/db* mice, or sections stained with anti-Mac2 antibodies from **I-L)** C57BL/6 mice or **M-P)** *db/db* mice, on the indicated diets. Bars are 0.1 mm.

**Fig 8:**
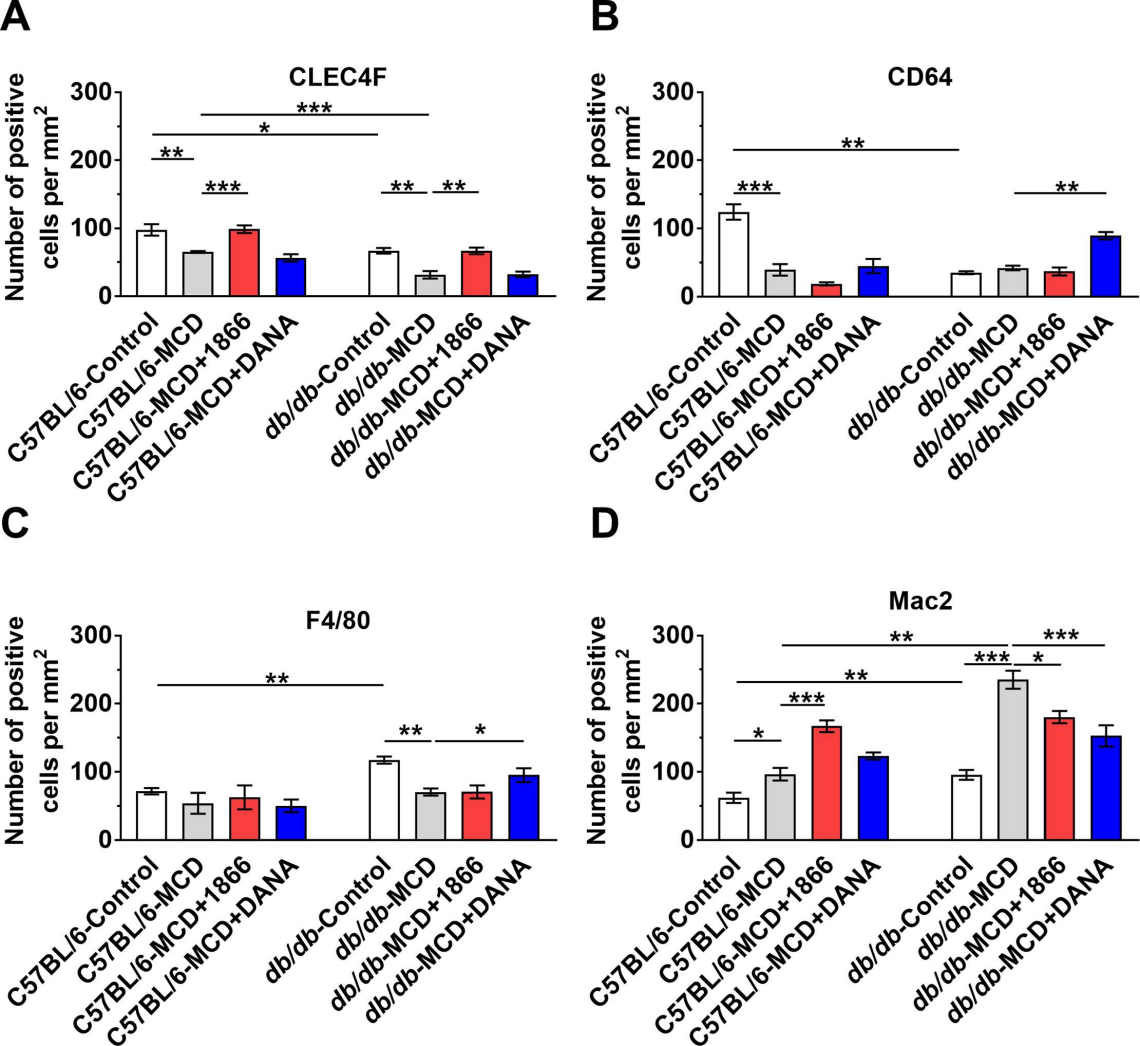
1866 or DANA injections reduce MCD diet-induced changes in liver macrophages. Quantification of **A)** CLEC4F, **B)** CD64, **C)** F4/80 or **D)** Mac2 positive cells. Values are mean ± SEM, n = 3–5 mice per group. * indicates p < 0.05, ** p < 0.01, and ** p < 0.001 (one-way ANOVA, Sidak’s test).

NASH and the MCD mouse model are associated with liver inflammation, especially an increase in macrophages [32, 70, 86, 87]. Liver macrophages are a diverse group of cells including embryonically derived Kupffer cells and recruited monocyte-derived macrophage subsets that may either promote or inhibit liver damage and repair [88–92]. For C57BL/6 mice, as observed previously [32, 93], the MCD diet reduced numbers of CLEC4F positive Kupffer cells (Figs 7A, 7B and 8A). 1866 reversed the loss of CLEC4F positive cells (Figs 7A–7D and 8A). Compared to C57BL/6 mice, *db/db* mice on both control and MCD diet had reduced numbers of CLEC4F positive cells (Figs 7A, 7B, 7E, 7F and 8A). For *db/db* mice, 1866 also reversed the MCD diet-induced loss of CLEC4F positive cells (Figs 7E–7H, and 8A). These data suggest that for mice on the MCD diet, 1866 can increase CLEC4F positive cell numbers, either by inhibiting cell death, or promoting renewal of CLEC4F positive cells by proliferation or differentiation [14].

For C57BL/6 mice, the MCD diet reduced numbers of CD64 but not F4/80 positive resident liver macrophages [94–97] (Figs 8B and 8C and S3 Fig). 1866 or DANA did not affect these numbers. Compared to C57BL/6 mice, *db/db* mice on a control diet had reduced numbers of CD64 but increased numbers of F4/80 positive cells (Fig 8B and 8C and S3A–S3H Fig). For *db/db* mice, the MCD diet reduced numbers of F4/80 but not CD64 positive cells (Fig 8B and 8C and S3I–S3P Fig), and DANA increased the numbers of CD64 and F4/80 positive cells (Fig 8B and 8C).

For C57BL/6 mice, as observed previously [32, 70], the MCD diet increased numbers of Mac2 positive recruited macrophages [94–97] in the liver (Figs 7I, 7J and 8D). 1866 further increased the numbers of Mac2 positive cells (Figs 7I–7L and 8D). Compared to C57BL/6 mice, *db/db* on both control and MCD diet had increased numbers of Mac2 positive cells (Figs 7I, 7J, 7M, 7N and 8D). For *db/db* mice, 1866 and DANA reduced MCD diet-increased numbers of Mac2 positive cells (Figs 7M–7P and 8D). These data suggest that MCD diet-induced changes in liver macrophage numbers are altered by 1866 and DANA, suggesting a complex interaction between liver macrophages and the effect of the MCD diet.

To determine if the increase in the number of Mac2 positive cells was characteristic of a general inflammatory response, we also stained liver tissues with anti-MRP8 antibodies to detect neutrophils [98, 99]. Compared to the number of macrophages in the liver (Fig 8), there were few MRP8 positive cells in liver tissue of C57BL/6 or *db/db* mice (S4 Fig). In C57BL/6 mice we did not detect any significant differences in the number of MRP8 positive cells on regular or MCD diet (, or treated with 1866 or DANAS4A–S4D and S4I Fig). Compared to C57BL/6 mice, *db/db* on the MCD diet had a small increase in the number of MRP8 positive cells, and 1866 reduced these numbers (S4E–S4I Fig). These data suggest that MCD diet-induced changes in macrophages is not a generalized effect on all myeloid cells.

**Fig 9:**
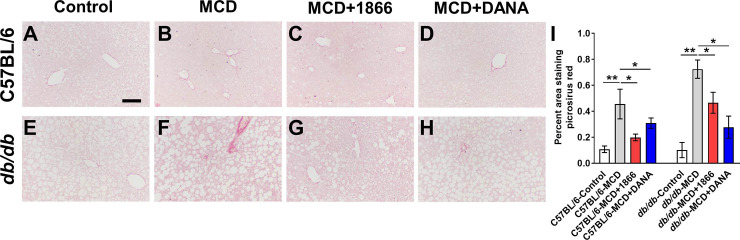
1866 and DANA reduce MCD diet-induced liver fibrosis. **A-D)** C57BL/6 and **E-H)** *db/db* mice on **A and E)** control diet, **B and F)** MCD diet, **C and G)** MCD+1866, or **D and H)** MCD + DANA. Images show representative liver sections stained with sirius red. Bar is 0.1 mm. **I)** Quantification of fibrosis in liver sections. Values are mean ± SEM, n = 3–5 mice per group. * indicates p<0.05, ** p < 0.01 (one-way ANOVA, Sidak’s test).

NASH and the MCD mouse model are associated with liver fibrosis [8, 20, 34]. Compared with control diet mice (Fig 9A and 9E), C57BL/6 and *db/db* mice on the MCD diet had increased staining with sirius red (Fig 9B and 9F), and treatment with 1866 or DANA reduced sirius red staining (Fig 9C, 9D and 9G–9I). These data suggest that 1866 and DANA can attenuate some of the effects of MCD diet on liver fibrosis.

## Discussion

Although the differences in the response to 1866 or DANA between C57BL/6 and *db/db* mice might be specific for only one genotype or one compound, we found that injections of 1866 and DANA attenuate multiple features of MCD diet-induced changes in adipose tissue and liver of mice. 1866 or DANA had little effect on glucose levels in the C57BL/6 or *db/db* MCD diet models. For C57BL/6 mice on the MCD diet, 1866 and DANA reversed the decrease in white fat weight and increases in Mac2 positive cells in white fat, and 1866 also increased brown fat weight. In *db/db* mice, 1866 and DANA reduced MCD diet induced increases in crown like structures in the white fat. In the liver, the MCD diet induces both steatosis and inflammation. In *db/db* but not C57BL/6 mice, 1866 also reduced liver steatosis. Both the MCD diet and the dysregulation of metabolism in the *db/db* mouse cause a decrease in the numbers of CLEC4F-positive Kupffer cells, but 1866 reversed these effects. Both the MCD diet and the *db/db* mouse lead to the loss of liver resident CD64 positive cells and an increase in F4/80 macrophages, and DANA reversed these effects. For both C57BL/6 and *db/db* mice on the MCD diet, there were increased Mac2 positive cells in the livers, and in the *db/db* mice, both 1866 and DANA reduced the number of Mac2 positive cells. These results indicate that 1866 and DANA could be useful to protect adipocytes, albeit not in the presence of diabetes, and that 1866 could be useful for steatosis, but only when there is diabetes. In addition, the changes in the local density of 3 different adipose tissue macrophages and 4 different liver macrophages cell types did not correlate with effects on adipocyte size or liver steatosis.

We have previously shown that 1866 can ameliorate inflammation in multiple animal models [47, 54]. 1866 appears to act, at least in part by stimulating IL-10 production by a variety of cell types including adipocytes, reducing serum levels of pro-inflammatory cytokines such as IFN-γ, and inhibiting adipocyte differentiation in vitro [47, 54]. These effects are dependent on CD209 expression, which appears to be limited to dendritic cells, monocyte/macrophage subsets, and neutrophils, including CD209 expressing cells present in the liver [47, 100]. DANA inhibits all mammalian sialidases [58, 101], and can also inhibit inflammation in multiple animal models [59, 101, 102]. Sialidases remove the terminal sialic acid residues from glycoproteins, which for proteins such as SAP and IgG, reverses the proteins anti-inflammatory properties [47, 103]. DANA can also inhibit a sialidase-TGF-β positive feedback loop, where NEU3 upregulates active TGF-β1 by releasing TGF-β1 from its latent inactive form by desialylating latency associated glycopeptide (LAP) [59, 101]. This suggests that both 1866 and DANA may act on multiple pathways to modulate adipose tissue and liver macrophage populations and steatosis in mice.

The accumulation of immune cells, especially monocyte-derived macrophages, in both adipose tissue and liver is thought to promote the progression from NAFLD to NASH and fibrosis, whereas tissue resident macrophages are generally thought to be homeostatic [22, 25, 82, 104–106]. However, not all obese individuals develop NAFLD or NASH, and NAFLD occurs in lean individuals [107, 108]. In addition, many anti-inflammatory strategies have had only modest effects at modulating insulin resistance and glucose regulation, lipid accumulation in the liver, or the progression from NAFLD to NASH and fibrosis [3, 109]. These results indicate that although steatosis is considered an initiator of liver inflammation, steatosis and inflammation could be viewed as independent or parallel processes. We found that in the mouse high fat diet (HFD) model of inflammation and steatosis of adipose tissue and liver, DANA, but not 1866, attenuates the increase in glucose levels after glucose injection [54, 102]. However, in the HFD model, both DANA and 1866 decrease adipose tissue and liver inflammation and steatosis [54, 102]. These results suggest that either the degree or length of insult, such as the relatively mild but long-term insult of HFD compared to the more aggressive short-term MCD diet, the involvement of distinct immune and non-immune cell types in promoting or inhibiting disease progression, or dysregulation of the different pathways that lead to lipid accumulation in adipose and liver tissue, may differentially induce accumulation of immune cells in adipose or liver tissue, and may help to explain the spectrum or subtypes of NAFLD [97, 110–115].

The levels of sialidases in HFD-induced obesity is unclear, with up or down regulation of different sialidase mRNA and proteins in different tissues in different rodent models [116–118]. We have previously found that for lung and adipose tissue, antibody staining for all four sialidases was not significantly different between regular and HFD fed C57BL/6 mice, but DANA injections did reduce staining for NEU3 in adipose tissue in mice on regular diet [102]. In the livers of C57BL/6 mice, DANA increased NEU1 and NEU3 antibody staining, but DANA reduced NEU3 staining in HFD mice [102]. In *Neu3* knock out mice, NEU1, NEU2, and NEU4 protein levels are reduced in lung, adipose and liver tissue [60, 102]. We have also shown that the expression of sialidase mRNA levels are not a good predictor of protein expression, as TGF-β can modulate NEU3 protein levels by decreasing NEU3 degradation and by increasing the translation of NEU3 mRNA, explaining the apparent paradox of high levels of NEU3 protein in pulmonary fibrosis without a concomitant increase in the expression of NEU3 mRNA [119]. This suggest that sialidase expression is complex, and individual tissues or cells may differentially express different sialidase proteins, depending on the environmental conditions.

The MCD diet mimics the steatosis and inflammation associated with NASH, without the systemic changes and weight gain associated with obesity [18, 20, 32]. The accumulation of Mac2 positive macrophages in adipose tissue and liver, and the ability of 1866 and DANA to differentially modulate their numbers, suggest that these two different compounds have distinct but overlapping effects on macrophage accumulation. 1866 is a DC-SIGN/CD209 ligand [47, 120], which is expressed on both immune and non-immune cells in adipose tissue and liver [80, 94, 95, 121–123]. We previously found that 1866 can decrease high fat diet induced adipose tissue and liver inflammation [54], and the observation that MCD diet-induced inflammation is also decreased by 1866 suggests that 1866 could act as a general anti-inflammatory compound. DANA acts by inhibiting sialidases [58, 59], and monocyte-macrophages can express all four sialidases as they differentiate and depending on stimuli they detect [59, 124, 125]. The observation that MCD diet-induced increases in Mac2 positive cells in adipose tissue and liver, and loss of F4/80 positive cells in the liver, were reversed by DANA suggests that sialidases regulate some of the macrophage populations in these tissue [25, 82, 89, 104, 126].

The lack of methionine and choline in the diet, which are necessary for hepatic mitochondrial β-oxidation (which is necessary to convert excess fatty acids into acetyl CoA), and very low-density lipoprotein (VLDL) synthesis (which are necessary to transport lipids between tissues), leads to liver steatosis [20, 33]. We observed differential effects of 1866 and DANA on MCD diet-induced liver steatosis. In *db/db* mice, the MCD diet generates an exaggerated hepatic steatosis, and this was reversed by 1866 but not DANA. This observation suggests that either sialidases are not important in the steatotic process, or that 1866 acts presumably on the CD209 receptor present on some cell type(s) in the liver to regulate the steatotic response. The CD209 receptor is upregulated in adipose tissue of NASH patients [127] and we previously found that 1866 modulates HFD-induced changes in liver steatosis [54]. How 1866 regulates steatosis, and on which cells 1866 acts, is unknown.

Together, these data suggest that CD209 and sialidases function to regulate adipose and liver tissue inflammation, and that CD209 ligands and sialidase inhibitors are potential therapeutics for steatosis and liver inflammation.

## Supporting information

S1 Fig Organ weights. C57BL/6 and *db/db* mice were transferred to methionine and choline sufficient (Control) or methionine and choline deficient (MCD) diet at day 0 and were injected every 48 hours with buffer, 1866, or DANA. At day 21 for **A)** C57BL/6 or **B)** 28 days for *db/db* mice, post-euthanasia tissues were weighted. Absolute body and organ weights for **C)** C57BL/6 and **D)** *db/db* mice. x2 indicates both kidneys were weighed together. Values are mean ± SEM, n = 3–5 mice per group. * indicates p < 0.05, **p < 0.01, and *** p < 0.001 (one-way ANOVA, Sidak’s test). † indicates p < 0.05, †† p< 0.01, and ††† p<0.001 comparing C57BL/6 and *db/db* mice on control diet (t-test). # indicates p < 0.05 and ### p<0.001 comparing C57BL/6 and *db/db* mice on MCD diet (t-test).
(TIF)

S2 Fig Quantification of serum cytokine levels. Sera from **A)** C57BL/6 or **B)** *db/db* mice were assessed for the indicated cytokines. Values are means ± SEM from 3–5 mice.
(TIF)

S3 Fig MCD diet-induced changes in CD64 and F4/80 positive liver macrophages. Representative images of liver sections stained with anti-CD64 antibodies from **A-D)** C57BL/6 mice or **E-H)** or *db/db* mice, or sections stained with anti-F4/80 antibodies from **I-L)** C57BL/6 mice or **M-P)** *db/db* mice, on the indicated diets. Bars are 0.1 mm.
(TIF)

S4 Fig 1866 injections reduce MCD diet-induced changes in liver neutrophils. Representative images of liver sections stained with anti-MRP8 antibodies from **A-D)** C57BL/6 mice or **E-H)** or *db/db* mice. **I)** Quantification of MRP8 positive cells. Values are mean ± SEM, n = 3–5 mice per group. * indicates p < 0.05 (one-way ANOVA, Sidak’s test) or # p < 0.05 (t test).
(TIF)
