# Supplementary figures and images for: A CD209 ligand and a sialidase inhibitor differentially modulate adipose tissue and liver macrophage populations and steatosis in mice on the Methionine and Choline-Deficient (MCD) diet
Source: PLoS One. 2020 Dec 30;15(12):e0244762. doi: 10.1371/journal.pone.0244762 (PMC7773271; doi:10.1371/journal.pone.0244762)

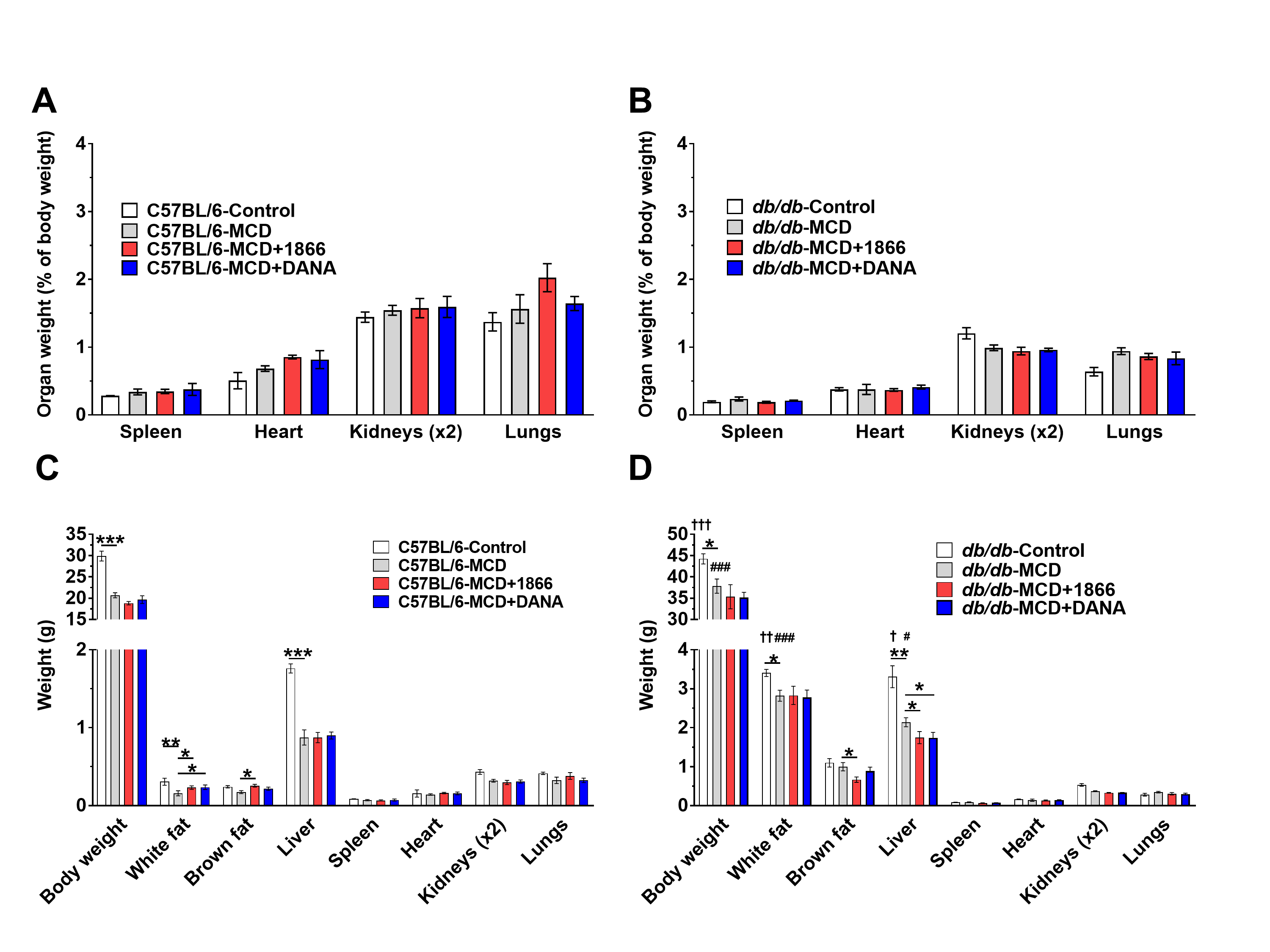

Supplement: S1 Fig — C57BL/6 and db/db mice were transferred to methionine and choline sufficient (Control) or methionine and choline deficient (MCD) diet at day 0 and were injected every 48 hours with buffer, 1866, or DANA. At day 21 for A) C57BL/6 or B) 28 days for db/db mice, post-euthanasia tissues were weighted. Absolute body and organ weights for C) C57BL/6 and D) db/db mice. x2 indicates both kidneys were weighed together. Values are mean ± SEM, n = 3–5 mice per group. * indicates p < 0.05, **p < 0.01, and *** p < 0.001 (one-way ANOVA, Sidak’s test). † indicates p < 0.05, †† p< 0.01, and ††† p<0.001 comparing C57BL/6 and db/db mice on control diet (t-test). # indicates p < 0.05 and ### p<0.001 comparing C57BL/6 and db/db mice on MCD diet (t-test). (TIF) [file pone.0244762.s001.TIF]

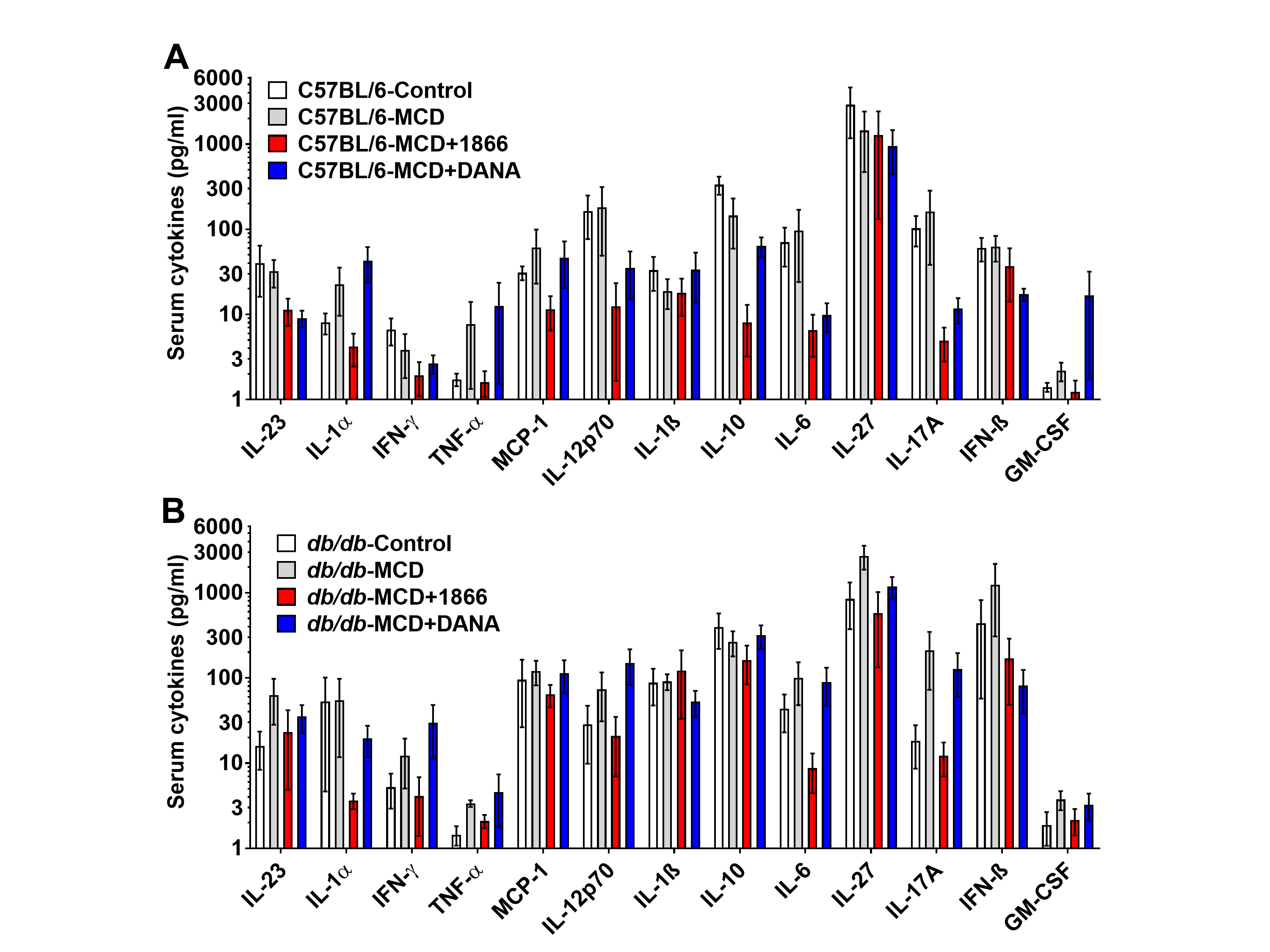

Supplement: S2 Fig — Sera from A) C57BL/6 or B) db/db mice were assessed for the indicated cytokines. Values are means ± SEM from 3–5 mice. (TIF) [file pone.0244762.s002.TIF]

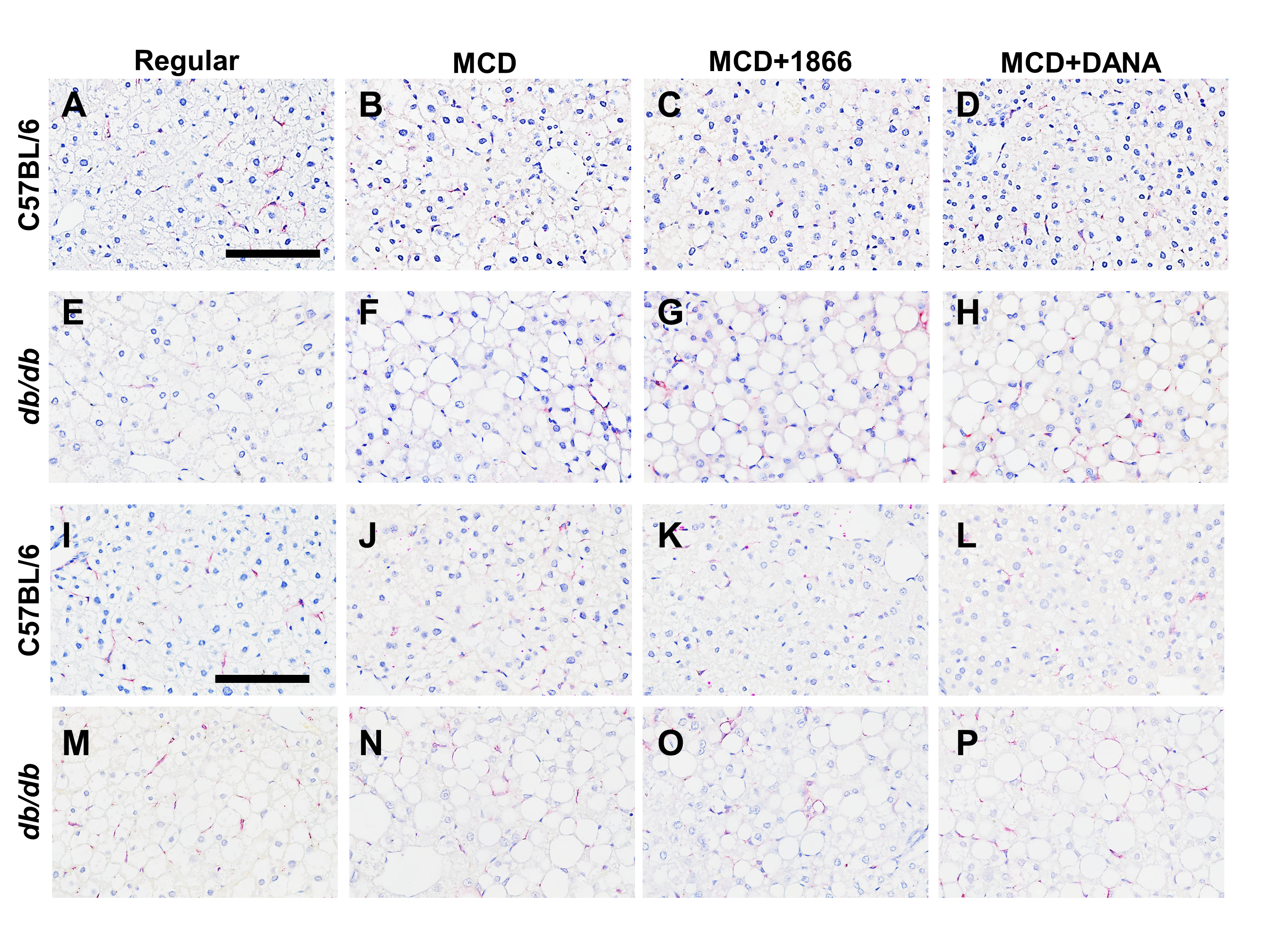

Supplement: S3 Fig — Representative images of liver sections stained with anti-CD64 antibodies from A-D) C57BL/6 mice or E-H) or db/db mice, or sections stained with anti-F4/80 antibodies from I-L) C57BL/6 mice or M-P) db/db mice, on the indicated diets. Bars are 0.1 mm. (TIF) [file pone.0244762.s003.TIF]

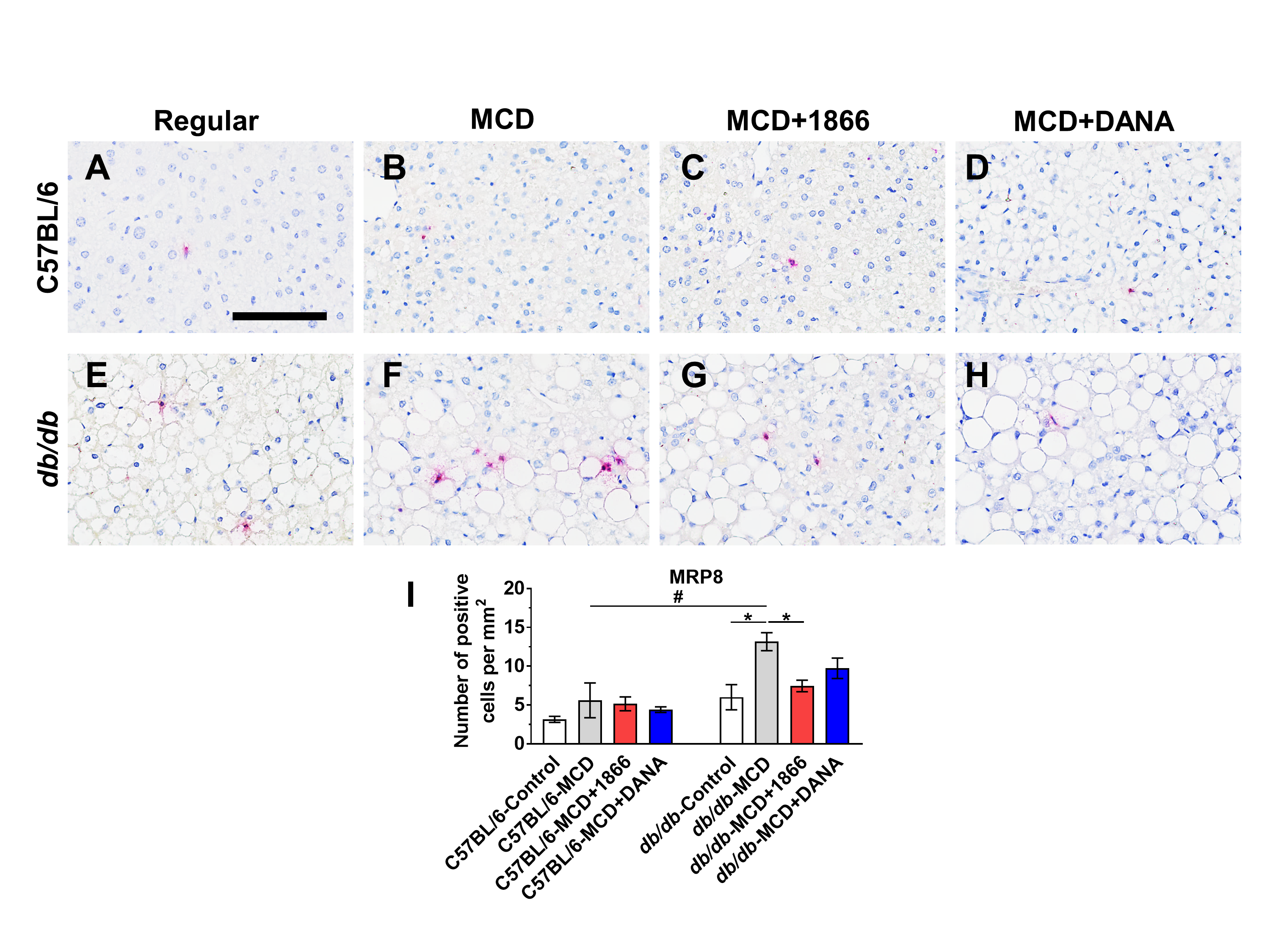

Supplement: S4 Fig — Representative images of liver sections stained with anti-MRP8 antibodies from A-D) C57BL/6 mice or E-H) or db/db mice. I) Quantification of MRP8 positive cells. Values are mean ± SEM, n = 3–5 mice per group. * indicates p < 0.05 (one-way ANOVA, Sidak’s test) or # p < 0.05 (t test). (TIF) [file pone.0244762.s004.TIF]
